# Renal Dysfunction during Tenofovir Use in a Regional Cohort of HIV-Infected Individuals in the Asia-Pacific

**DOI:** 10.1371/journal.pone.0161562

**Published:** 2016-08-25

**Authors:** Junko Tanuma, Awachana Jiamsakul, Abhimanyu Makane, Anchalee Avihingsanon, Oon Tek Ng, Sasisopin Kiertiburanakul, Romanee Chaiwarith, Nagalingeswaran Kumarasamy, Kinh Van Nguyen, Thuy Thanh Pham, Man Po Lee, Rossana Ditangco, Tuti Parwati Merati, Jun Yong Choi, Wing Wai Wong, Adeeba Kamarulzaman, Evy Yunihastuti, Benedict LH Sim, Winai Ratanasuwan, Pacharee Kantipong, Fujie Zhang, Mahiran Mustafa, Vonthanak Saphonn, Sanjay Pujari, Annette H. Sohn, TREAT Asia HIV Observational Databases (TAHOD)

**Affiliations:** 1 AIDS Clinical Center, National Center for Global Health and Medicine, Tokyo, Japan; 2 The Kirby Institute, UNSW Australia, Sydney, Australia; 3 Institute of Infectious Diseases, Pune, India; 4 The Netherland Australia Thailand Research Collaboration/Thai Red Cross AIDS Research Centre, Faculty of Medicine, Chulalongkorn University, Bangkok, Thailand; 5 Department of Infectious Disease and Communicable Disease Centre, Tan Tock Seng Hospital, Singapore, Singapore; 6 Faculty of Medicine, Ramathibodi Hospital, Mahidol University, Bangkok, Thailand; 7 Research Institute for Health Sciences, Chiang Mai, Thailand; 8 Chennai Antiviral Research and Treatment Clinical Research Site, Y.R. Gaitonde Centre for AIDS Research and Education, Chennai, India; 9 National Hospital of Tropical Diseases, Hanoi, Vietnam; 10 Infectious Disease Department, Bach Mai Hospital, Hanoi, Vietnam; 11 Department of Medicine, Queen Elizabeth Hospital, Hong Kong, China; 12 Department of Health, Research Institute for Tropical Medicine, Manila, Philippines; 13 Faculty of Medicine, Udayana University & Sanglah Hospital, Bali, Indonesia; 14 Division of Infectious Diseases, Department of Internal Medicine, Yonsei University College of Medicine, Seoul, South Korea; 15 Infectious Diseases and AIDS Unit, Department of Medicine, Taipei Veterans General Hospital, Taipei, Taiwan; 16 Faculty of Medicine, University Malaya Medical Centre, Kuala Lumpur, Malaysia; 17 Faculty of Medicine, University of Indonesia, Cipto Mangunkusumo Hospital, Jakarta, Indonesia; 18 Department of Medicine, Hospital Sungai Buloh, Sungai Buloh, Malaysia; 19 Faculty of Medicine, Siriraj Hospital, Mahidol University, Bangkok, Thailand; 20 Department of Medicine, Chiangrai Prachanukroh Hospital, Chiang Rai, Thailand; 21 Beijing Ditan Hospital, Capital Medical University, Beijing, China; 22 Medical Department, Hospital Raja Perempuan Zainab II, Kota Bharu, Malaysia; 23 National Center for HIV/AIDS, Dermatology & STDs, University of Health Sciences, Phnom Penh, Cambodia; 24 Institute of Infectious Diseases, Pune, India; 25 TREAT Asia, amfAR – The Foundation for AIDS Research, Bangkok, Thailand; University of Sao Paulo Medical School, BRAZIL

## Abstract

**Background:**

In resource-limited settings, routine monitoring of renal function during antiretroviral therapy (ART) has not been recommended. However, concerns for tenofovir disoproxil fumarate (TDF)-related nephrotoxicity persist with increased use.

**Methods:**

We investigated serum creatinine (S-Cr) monitoring rates before and during ART and the incidence and prevalence of renal dysfunction after starting TDF by using data from a regional cohort of HIV-infected individuals in the Asia-Pacific. Time to renal dysfunction was defined as time from TDF initiation to the decline in estimated glomerular filtration rate (eGFR) to <60 ml/min/1.73m^2^ with >30% reduction from baseline using the Chronic Kidney Disease Epidemiology Collaboration (CKD-EPI) equation or the decision to stop TDF for reported TDF-nephrotoxicity. Predictors of S-Cr monitoring rates were assessed by Poisson regression and risk factors for developing renal dysfunction were assessed by Cox regression.

**Results:**

Among 2,425 patients who received TDF, S-Cr monitoring rates increased from 1.01 to 1.84 per person per year after starting TDF (incidence rate ratio 1.68, 95%CI 1.62–1.74, p <0.001). Renal dysfunction on TDF occurred in 103 patients over 5,368 person-years of TDF use (4.2%; incidence 1.75 per 100 person-years). Risk factors for developing renal dysfunction included older age (>50 vs. ≤30, hazard ratio [HR] 5.39, 95%CI 2.52–11.50, p <0.001; and using PI-based regimen (HR 1.93, 95%CI 1.22–3.07, p = 0.005). Having an eGFR prior to TDF (pre-TDF eGFR) of ≥60 ml/min/1.73m^2^ showed a protective effect (HR 0.38, 95%CI, 0.17–0.85, p = 0.018).

**Conclusions:**

Renal dysfunction on commencing TDF use was not common, however, older age, lower baseline eGFR and PI-based ART were associated with higher risk of renal dysfunction during TDF use in adult HIV-infected individuals in the Asia-Pacific region.

## Introduction

The widespread use of antiretroviral therapy (ART) has brought a marked decline in mortality and morbidity of HIV-infected individuals, but concerns have grown regarding the emergence of other chronic diseases associated with extended life expectancies coupled with the long-term effects of HIV disease and its treatment. One of the serious non-AIDS conditions which have increased mortality in the post-ART era is chronic kidney disease (CKD) [1, 2]. Although rapidly progressive HIV-associated nephropathy (HIVAN) has less frequently been seen, nephrotoxicity due to some antiretrovirals (ARV), including tenofovir disoproxil fumarate (TDF), has been well documented [3–7].

TDF is rapidly becoming one of the most widely used ARVs in the world [8–10]. Although the mechanism of TDF-related nephrotoxicity has not been fully elucidated, it presents with decreased glomerular filtration rate (GFR) and proximal tubular dysfunction [11]. TDF nephrotoxicity may be partly irreversible; therefore early detection of renal dysfunction is a key element of the clinical management [12, 13]. The HIV Medicine Association of the Infectious Diseases Society of America (IDSA) recommends twice yearly monitoring of estimated GFR (eGFR), serum phosphate and urinalysis while receiving TDF [7, 14].

On the other hand, frequent laboratory monitoring of serum creatinine (S-Cr) may not be practical in resource-limited settings, and the World Health Organization (WHO) has yet to recommend routine S-Cr testing before and during ART [10]. As TDF use has expanded in resource-limited settings, there are limited data on how often renal function is being monitored and the extent of associated nephrotoxicity being observed [10].

In this analysis, we evaluated the frequencies of S-Cr measurement before and during TDF use, and the incidence and factors of renal dysfunction while on TDF in a large prospective cohort in the Asia-Pacific region: the TREAT Asia HIV Observational Database (TAHOD) [15].

## Methods

Two analyses were conducted based on data collected in TAHOD [15]. Briefly, TAHOD is an observational study of patients with HIV involving 22 adult treatment centers in 12 countries and territories of varying income levels in Asia. The study was established in 2003 and aims to assess HIV disease natural history in treated and untreated patients in the region. Retrospective and prospective data is collected at each site. Data is transferred to the data management center at the Kirby Institute, Sydney, Australia, twice annually in March and September.

### Analysis (i): To determine frequencies of S-Cr monitoring before and during TDF use

In this analysis, we included TAHOD patients who had ever received TDF as part of an ART regimen consisting of at least three ARVs. Factors associated with rates of S-Cr monitoring (S-Cr rates) were analyzed using a Poisson regression model with random effects on the patient to account for repeated measurements of S-Cr in individual patients. Analysis time began from ART initiation and was censored when TDF was discontinued for more than seven days, at death or the last follow-up date, whichever occurred first. Patients who re-started TDF after seven days did not re-enter the analysis risk set. Time-fixed covariates included in the model were age, weight, CD4 count, viral load (VL) and hepatitis B/C co-infection at ART initiation, mode of HIV exposure, initial ART regimen, prior AIDS defining illnesses (Centers for Disease Control and Prevention [CDC] Category C), pre-ART eGFR, and World Bank country income level [16]. Time-updated covariates were TDF use, indinavir (IDV) use, high fasting glucose level (FGL) and high blood pressure. High FGL was defined as a single FGL ≥7 mmol/L or ≥126 mg/dL. High blood pressure was defined as a single systolic >140 mmHg or diastolic >90 mmHg measurement.

Trends in crude rates of S-Cr testing for each year on TDF were also explored using Poisson regression with follow-up time beginning from TDF initiation.

### Analysis (ii): To determine factors associated with time to renal dysfunction on TDF

Patient selection followed the same criteria as per analysis (i). TDF-related renal toxicity was defined as having eGFR reduction of at least 30% of the baseline value and <60 ml/min/1.73m^2^, or having ART stopped for reported renal toxicity. The eGFR value used to calculate the percentage decline was the value within six months prior to and closest to the date of TDF initiation. In order to include patients without pre-TDF S-Cr assessment, the first S-Cr after TDF initiation was used as the initial eGFR. The eGFR was calculated according to the Chronic Kidney Disease Epidemiology Collaboration (CKD-EPI) equation [17]. The analysis risk time began at TDF initiation rather than from the time of overall ART initiation. To account for censored observations, time to renal dysfunction was analyzed using a Cox regression model and stratified by site. Patients were censored at TDF discontinuation of more than seven days, the last follow-up date or death. Covariates included in the model were age at TDF initiation, sex, mode of HIV exposure, pre-TDF weight, pre-TDF eGFR, pre-TDF VL and CD4 count, TDF combination, prior ART exposure, hepatitis B/C co-infection, prior AIDS illnesses, S-Cr assessment in the previous six months, indinavir use, high FGL, high blood pressure, and ART adherence. S-Cr assessment in the previous six months was coded as a time-updated binary variable where “Yes” referred to having at least one S-Cr assessment in the previous 6-month interval, and “No” meant having no assessment in that time period. To construct this covariate, time was calculated as discrete 6-monthly intervals from TDF initiation (e.g., first six months, second six months). Therefore, if a patient’s total follow-up time were currently seven months from TDF initiation, the S-Cr assessment covariate for this patient would refer to the time period between month one to six. If the follow-up time were 11 months, the S-Cr covariate would still refer to month one to six. As such, the S-Cr covariate did not vary continuously, but discretely according to the time interval from TDF initiation. ART adherence was collected from the self-reported visual analogue scale (VAS) [18], and coded as <95%, ≥95% or missing.

All regression models were fitted using backward stepwise selection process. Variables significant in the univariate analysis at Wald’s test p <0.10 were chosen for inclusion in the multivariate model. Variables significant at p <0.05 were considered statistically significant in the final multivariate model. All data management and statistical analyses were performed using SAS software version 9.3 (SAS Institute Inc., Cary, NC, USA) and STATA software version 12.1 (STATA Corp., College Station, TX, USA).

Ethics approval for the TAHOD study design, methods, and consent procedures, was granted by the University of New South Wales, Human Research Ethics Committee. Site-specific study governance was granted by site-relevant institutional review boards. Written informed consent was not sought in TAHOD unless required by a site’s local institutional review board. Informed consent was waived at some sites as information is collected via an anonymous case report form. All study procedures were developed in accordance with the revised Helsinki Declaration.

## Results

The analysis included a total of 2,425 patients from Cambodia, China (including Hong Kong SAR), India, Indonesia, Japan, Malaysia, the Philippines, Singapore, South Korea, Taiwan, Thailand and Vietnam. Patients initiated ART between 1996 and 2013 and contributed to 13,019 person years on ART and 5,368 years on TDF use. Table 1 shows patient characteristics of these patients before ART initiation, and the number of patients with ≥2 average S-Cr assessments per year. Of the 2,425 patients, 69% were male with heterosexual exposure as the reported exposure category for HIV infection. The median age was 35 years (interquartile range [IQR]: 30–42), median VL was 100,000 copies/mL (IQR: 34,388–270,000) and median CD4 count was 114 cells/μL (IQR: 39–214). Initial ART combinations consisted mainly of nucleoside reverse transcriptase inhibitors (NRTI), plus non-NRTIs (NNRTI; 83%). More than 60% had no hepatitis B or C co-infections or prior AIDS illnesses. Six hundred and twenty seven of the 2,425 patients (26%) had ≥2 S-Cr assessments per year. The proportion was relatively higher after TDF initiation than prior to TDF use (38% vs. 18%, p<0.001). These characteristics are also reported by country in S1 Table.

**Table 1:** Baseline demographics of patients who have ever received TDF.

	Total (%)	^a^Number with average S-Cr assessments ≥2 per year ^b^(%)
	2,425 (100)	627 (25.9)
**Age at ART initiation (years)**	median 35, IQR (30–42)	
≤30	680 (28.0)	151 (22.2)
31–40	1,043 (43.0)	256 (24.5)
41–50	499 (20.6)	149 (29.9)
>50	203 (8.4)	71 (35.0)
**Sex**		
Male	1,668 (68.8)	498 (29.9)
Female	757 (31.2)	129 (17.0)
**Mode of HIV Exposure**		
Heterosexual contact	1,671 (68.9)	317 (19.0)
Homosexual contact	452 (18.6)	203 (44.9)
Injecting drug use	164 (6.8)	39 (23.8)
Other/unknown	138 (5.7)	68 (49.3)
**Pre-ART weight (kg)**	median = 54.9, IQR (48–63.8)	
≤55	823 (33.9)	162 (19.7)
>55	732 (30.2)	244 (33.3)
Missing	870 (35.9)	221 (25.4)
**Pre-ART viral load (copies/mL)**	median = 100,000, IQR (34,388–270,000)	
≤100,000	599 (24.7)	217 (36.2)
>100,000	586 (24.2)	225 (38.4)
Missing	1,240 (51.1)	185 (14.9)
**Pre-ART CD4 (cells/μL)**	median = 114, IQR (39–214)	
≤50	634 (26.1)	129 (20.3)
51–100	311 (12.8)	66 (21.2)
101–200	519 (21.4)	155 (29.9)
>200	561 (23.1)	204 (36.4)
Missing	400 (16.5)	73 (18.3)
**Initial ART Regimen**		
NRTI+NNRTI	2,014 (83.1)	402 (20.0)
NRTI+PI	365 (15.1)	211 (57.8)
Other	46 (1.9)	14 (30.4)
**Hepatitis B co-infection**		
Negative	1,593 (65.7)	468 (29.4)
Positive	334 (13.8)	102 (30.5)
Not tested	498 (20.5)	57 (11.4)
**Hepatitis C co-infection**		
Negative	1,547 (63.8)	472 (30.5)
Positive	263 (10.8)	58 (22.1)
Not tested	615 (25.4)	97 (15.8)
**Previous AIDS**		
No	1,470 (60.6)	403 (27.4)
Yes	955 (39.4)	224 (23.5)
**Country Income Level**		
Low + Lower Middle	898 (37.0)	137 (15.3)
Upper Middle	1,196 (49.3)	288 (24.1)
High	331 (13.6)	202 (61.0)
**Pre-ART eGFR (ml/min per 1.73 m**^**2)**^		
<60	25 (1.0)	15 (60.0)
≥60	1166 (48.1)	470 (40.3)
Missing	1234 (50.9)	142 (11.5)
^c^**TDF use**		
Prior to TDF initiation	N/A	293 (17.9)
After TDF initiation	N/A	929 (38.3)

Note:

^a^—Average calculated by the total number of serum creatinine assessments divided by the total follow-up time for each patient.

^b^—Proportion of patients having two serum creatinine assessments divided by the total number of patients in the same category.

^c^—Time-updated variable. The same patient can be counted in both categories.

TDF, tenofovir disoproxil fumarate; S-Cr, serum creatinine; ART, antiretroviral therapy; IQR, interquartile range; NRTI, nucleos(t)ide reverse transcriptase inhibitor; NNRTI, non-nucleoside reverse transcriptase inhibitor; PI, protease inhibitor

### Analysis (i): To determine rates of S-Cr monitoring before and during TDF use

Table 2 shows results from a Poisson-random effects model for factors associated with S-Cr monitoring after ART initiation. The overall crude rate was 1.41 per person-year (/PY). The rate while receiving TDF was 1.89/PY, which was relatively higher than the rate before receiving TDF of 1.01/PY (p<0.001). The maximum follow-up time from ART initiation to the censoring date was 16.9 years, and the maximum time from TDF initiation was 9.6 years. The multivariate model showed that after adjusting for significant predictors, the incidence rate ratio (IRR) for S-Cr testing during TDF compared to prior to TDF use was 1.68 (95% confidence interval (CI) 1.62–1.74, p <0.001). Other factors associated with higher S-Cr monitoring frequencies were injecting drug use (IDU) compared to heterosexual mode of HIV exposure (IRR 1.22, 95% CI 1.08–1.38; p = 0.002), baseline VL >100,000 compared to ≤100,000 copies/mL (IRR 1.27, 95% CI 1.16–1.38; p <0.001), PI-based initial regimen compared to NNRTI-based regimen (IRR 1.27, 95% CI 1.16–1.38; p <0.001), World Bank upper middle-income countries (IRR 1.15, 95% CI 1.06–1.24; p <0.001) and high-income countries (IRR 2.05, 95% CI 1.83–2.30; p<0.001) compared to low- and lower middle-income countries, and high FGL compared to FGL below 7 mmol/L or 126 mg/dL (IRR 1.15, 95% CI 1.05–1.25; p = 0.002). Having eGFR prior to ART ≥60 ml/min/1.73m^2^ compared to <60 ml/mim/1.73m^2^ (IRR 0.72, 95%CI (0.54, 0.95); p = 0.019).

**Table 2:** Factors associated with rates of serum creatinine monitoring after ART initiation.

					Univariate			Multivariate		
	Person-years	Number S-Cr assessments	^c^Crude rate	95% CI	IRR	95% CI	p	IRR	95% CI	p
**Age at ART initiation (years)**							<0.001			
≤30	3769.9	4846	1.29	(1.25, 1.32)	1					
31–40	5877.8	8052	1.37	(1.34, 1.40)	1.06	(0.97, 1.15)	0.231			
41–50	2420.6	3903	1.61	(1.56, 1.66)	1.29	(1.16, 1.44)	<0.001			
51+	951.0	1608	1.69	(1.61, 1.78)	1.24	(1.07, 1.43)	0.003			
**Sex**										
Male	8719.6	12918	1.48	(1.46, 1.51)	1					
Female	4299.7	5491	1.28	(1.24, 1.31)	0.82	(0.76, 0.89)	<0.001			
**Mode of HIV Exposure**							<0.001			**0.009**
Heterosexual contact	9598.4	12463	1.30	(1.28, 1.32)	1			1		
Homosexual contact	2077.0	3675	1.77	(1.71, 1.83)	1.52	(1.38, 1.67)	<0.001	0.95	(0.87, 1.04)	0.304
Injecting drug use	657.5	1004	1.53	(1.44, 1.62)	1.18	(1.02, 1.37)	0.028	**1.22**	**(1.08, 1.38)**	**0.002**
Other/unknown	686.5	1267	1.85	(1.75, 1.95)	1.43	(1.23, 1.67)	<0.001	1.02	(0.89, 1.16)	0.800
**Pre-ART weight (kg)**										
>55	3227.3	5731	1.78	(1.73, 1.82)	1					
≤55	4106.6	5662	1.38	(1.34, 1.42)	0.80	(0.73, 0.89)	<0.001			
Missing	5685.4	7016	1.23	(1.21, 1.26)						
**Pre-ART viral load (copies/mL)**										
≤100000	2793.8	4967	1.78	(1.73, 1.83)	1			1		
>100000	2614.8	5370	2.05	(2.00, 2.11)	1.22	(1.10, 1.34)	<0.001	**1.27**	**(1.16, 1.38)**	**<0.001**
Missing	7610.7	8072	1.06	(1.04, 1.08)						
**Pre-ART CD4 (cells/μL)**							0.001			
≤50	3318.4	4702	1.42	(1.38, 1.46)	1					
51–100	1690.3	2161	1.28	(1.23, 1.33)	1.01	(0.89, 1.15)	0.848			
101–200	2784.5	4356	1.56	(1.52, 1.61)	1.12	(1.01, 1.25)	0.030			
>200	2427.3	4116	1.70	(1.64, 1.75)	1.21	(1.09, 1.35)	<0.001			
Missing	2798.8	3074	1.10	(1.06, 1.14)						
**Initial ART Regimen**							<0.001			**<0.001**
NRTI+NNRTI	10842.9	13985	1.29	(1.27, 1.31)	1			1		
NRTI+PI	1903.6	3983	2.09	(2.03, 2.16)	2.03	(1.84, 2.24)	<0.001	**1.27**	**(1.16, 1.38)**	**<0.001**
Other	272.8	441	1.62	(1.47, 1.77)	1.18	(0.91, 1.53)	0.219	0.83	(0.67, 1.02)	0.080
**Hepatitis B co-infection**										
Negative	8303.4	13200	1.59	(1.56, 1.62)	1					
Positive	1676.6	2676	1.60	(1.54, 1.66)	0.93	(0.84, 1.04)	0.190			
Not tested	3039.3	2533	0.83	(0.80, 0.87)						
**Hepatitis C co-infection**										
Negative	8389.9	13102	1.56	(1.54, 1.59)	1					
Positive	1138.6	1869	1.64	(1.57, 1.72)	0.97	(0.86, 1.10)	0.657			
Not tested	3490.9	3438	0.98	(0.95, 1.02)						
**Previous AIDS**										
No	7714.0	10758	1.39	(1.37, 1.42)	1					
Yes	5305.3	7651	1.44	(1.41, 1.47)	1.04	(0.96, 1.12)	0.359			
**Country Income Level**							<0.001			**<0.001**
Low + Lower Middle	4166.7	4326	1.04	(1.01, 1.07)	1			1		
Upper Middle	7078.0	10336	1.46	(1.43, 1.49)	1.39	(1.29, 1.51)	<0.001	**1.34**	**(1.24, 1.45)**	**<0.001**
High	1774.7	3747	2.11	(2.04, 2.18)	2.21	(1.97, 2.48)	<0.001	**2.05**	**(1.83, 2.30)**	**<0.001**
**pre-ART eGFR(ml/min per 1.73 m**^**2**^**)**										
<60	105.5	289	2.74	(2.44, 3.07)	1			1		
≥60	4539.7	9507	2.09	(2.05, 2.14)	0.75	(0.54, 1.03)	0.071	**0.72**	**(0.54, 0.95)**	**0.019**
Missing	8374.1	8613	1.03	(1.01, 1.05)						
^d^**Receiving TDF**										
No	7025.1	7098	1.01	(0.99, 1.03)	1			1		
Yes	5994.2	11311	1.89	(1.85, 1.92)	1.93	(1.86, 2.00)	<0.001	**1.68**	**(1.62, 1.74)**	**<0.001**
^d^**Receiving IDV**										
No	12706.8	17945	1.41	(1.39, 1.43)	1					
Yes	312.5	464	1.49	(1.36, 1.63)	0.95	(0.84, 1.07)	0.388			
^a^^,^^d^**High fasting glucose level**										
No	7566.6	13579	1.79	(1.76, 1.83)	1			1		
Yes	522.2	998	1.91	(1.80, 2.03)	1.11	(1.02, 1.21)	0.021	**1.15**	**(1.05, 1.25)**	**0.002**
Missing	4930.5	3832	0.78	(0.75, 0.80)						
^b^^,^^d^**High blood pressure**										
No	8047.3	11923	1.48	(1.46, 1.51)	1					
Yes	1429.3	2379	1.66	(1.60, 1.73)	1.08	(1.02, 1.14)	0.005			
Missing	3542.7	4107	1.16	(1.12, 1.20)						

Note:

^a^—high fasting glucose defined as ≥7 mmol/L or ≥126 mg/dL.

^b^—High blood pressure defined as systolic >140 mmHg, or diastolic >90 mmHg.

^c^—Crude rate, per person-year

^d^—Time-updated variables

Missing values were coded as a separate category and were excluded from test for heterogeneity.

S-Cr, serum creatinine; CI, confidential interval; IRR, incident rate ratio; ART, antiretroviral therapy; NRTI, nucleos(t)ide reverse transcriptase inhibitor; NNRTI, non-nucleoside reverse transcriptase inhibitor; PI, protease inhibitor; TDF, tenofovir disoproxil fumarate; IDV, indinavir.

Crude rates of S-Cr monitoring for each year on TDF are shown in Table 3. The highest rate of 2.06/PYs (95%CI 2.01–2.11) was observed in the first year on TDF. The rates were shown to have a decreasing trend (p <0.001) as time on TDF increased; however, rates remained above 1.00/PY across all years, suggesting that in this cohort S-Cr was measured, on average, at least annually while receiving TDF.

**Table 3:** Crude rates of serum creatinine monitoring for each year on TDF.

Year on TDF	Person-years of observation	Number of S-Cr assessments	Crude rate (per person-year)	95% CI
1st	3,041.02	6,260	2.06	(2.01, 2.11)
2nd	1,270.4	2,236	1.76	(1.69, 1.83)
3rd	824.6	1,445	1.75	(1.66, 1.85)
4th	515.41	781	1.52	(1.41, 1.63)
5th	220.93	361	1.63	(1.47, 1.81)
6th-10th	121.81	228	1.87	(1.64, 2.13)

TDF, tenofovir disoproxil fumarate; S-Cr, serum creatinine; CI, confidential interval.

### Analysis (ii): To determine factors associated with time to renal dysfunction on TDF

There were 649/2425 patients (27%) whose first S-Cr measurement used to estimate the baseline GFR occurred after TDF initiation. The median delay between TDF initiation and first S-Cr measurement in this subgroup of patients was 5 months (IQR: 1.4–13 months). The total number of patients with renal dysfunction during TDF use was 103/2425 (4.2%) and the crude toxicity rate was 1.75 per 100PYs. During a median time of follow-up of 2.07 (IQR 1.00–3.56 years, 89 met the eGFR criteria of a decline to 60 ml/min/1.73m^2^ with at least 30% reduction from the baseline. The remaining 14 patients were classified according to reported reasons for ARV discontinuation. Factors associated with time to renal dysfunction are shown in Table 4. The multivariate model showed that the older age group of >50 years (hazard ratio [HR] 5.39, 95%CI 2.52–11.50, p <0.001) had higher risk of developing renal dysfunction compared to those ≤30 years. Patients who took a PI-based regimen at the time of TDF initiation were almost twice as likely to have toxicity compared to those on an NNRTI-based regimen (HR 1.93, 95%CI 1.22–3.07, p = 0.005). Those with pre-TDF eGFR ≥60 ml/min/1.73m^2^ showed a protective effect (HR 0.38, 95%CI, 0.17–0.85, p = 0.018). ART adherence and S-Cr assessment in the previous 6-month interval were not associated with renal dysfunction.

**Table 4:** Factors associated with time to renal dysfunction during TDF use.

				Univariate			Multivariate		
	Person-years	Number of patients with renal dysfunction	^c^Crude rate	HR	95% CI	p	HR	95% CI	p
Total	5886.6	103	1.75						
Age at TDF initiation (years)						<0.001			
≤30	942.9	9	0.95	1			1		
31–40	2606.6	29	1.11	1.08	(0.51, 2.31)	0.836	1.09	(0.51, 2.34)	0.815
41–50	1679.7	26	1.55	1.50	(0.69, 3.26)	0.303	1.58	(0.73, 3.44)	0.248
51+	657.4	39	5.93	5.81	(2.75, 12.26)	<0.001	5.39	(2.52, 11.50)	<0.001
Sex									
Male	3983.7	78	1.96	1					
Female	1902.9	25	1.31	0.76	(0.47, 1.21)	0.249			
Mode of HIV Exposure						0.069			
Heterosexual contact	4120.9	81	1.97	1					
Homosexual contact	1202.7	10	0.83	0.35	(0.15, 0.79)	0.011			
Injecting drug use	291.7	4	1.37	0.75	(0.25, 2.23)	0.599			
Other/unknown	271.2	8	2.95	1.10	(0.51, 2.40)	0.800			
Pre-TDF Weight (kg)									
≤55	2012.8	35	1.74	1					
>55	2907.0	49	1.69	0.92	(0.59, 1.44)	0.716			
Missing	966.7	19	1.97						
Pre-TDF eGFR (ml/min 1.73 m^2^)									
<60	68.5	8	11.69	1			1		
≥60	3678.9	62	1.69	0.19	(0.09, 0.41)	<0.001	0.38	(0.17, 0.85)	0.018
Missing	2139.2	33	1.54						
Pre-TDF viral load (copies/mL)									
<5000	1915.3	31	1.62	1					
≥5000	2087.0	30	1.44	0.90	(0.52, 1.58)	0.718			
Missing	1884.2	42	2.23						
Pre-TDF CD4 (cells/μL)						0.208			
≤50	553.0	15	2.71	1					
51–100	409.2	6	1.47	0.54	(0.21, 1.41)	0.210			
101–200	1012.8	16	1.58	0.52	(0.25, 1.08)	0.078			
201+	3270.0	53	1.62	0.53	(0.29, 0.98)	0.043			
Missing	641.5	13	2.03						
TDF combination						0.011			
NNRTI	4210.9	66	1.57	1			1		
PI	1490.0	34	2.28	1.99	(1.25, 3.15)	0.003	1.93	(1.22, 3.07)	0.005
Other	185.7	3	1.62	2.13	(0.64, 7.11)	0.217	2.14	(0.64, 7.20)	0.217
Prior ARV exposure									
No	2154.9	24	1.11	1					
Yes	3731.6	79	2.12	1.80	(1.09, 2.99)	0.022			
Hepatitis B co-infection									
Negative	3824.0	60	1.57	1					
Positive	842.5	16	1.90	1.15	(0.65, 2.03)	0.632			
Not tested	1220.0	27	2.21	1.69	(0.90, 3.19)	0.103			
Hepatitis C co-infection									
Negative	3806.1	57	1.50	1					
Positive	469.1	10	2.13	1.56	(0.73, 3.32)	0.252			
Not tested	1611.4	36	2.23						
Previous AIDS									
No	3337.6	47	1.41	1					
Yes	2549.0	56	2.20	1.47	(0.98, 2.21)	0.064			
^d^Creatinine assessment in the previous 6 months									
No	2256.2	29	1.29	1					
Yes	3630.4	74	2.04	1.27	(0.78, 2.09)	0.336			
^a^^,^^d^High fasting glucose level									
No	937.3	16	1.71	1					
Yes	62.9	3	4.77	2.63	(0.74, 9.37)	0.135			
Not tested	4886.4	84	1.72						
^b^^,^^d^High blood pressure									
No	2097.9	33	1.57	1					
Yes	412.7	8	1.94	1.15	(0.53, 2.53)	0.722			
Not tested	3376.0	62	1.84						
^d^ART adherence									
<95%	30.8	1	3.25	1					
≥95%	2221.2	33	1.49	0.43	(0.06, 3.27)	0.414			
Missing	3634.6	69	1.90						

Note:

^a^—high fasting glucose defined as ≥7 mmol/L or ≥126 mg/dL.

^b^—High blood pressure defined as systolic >140 mmHg, or diastolic >90 mmHg.

^c^—Crude rate, per 100 person-years.

^d^—Time-updated variables.

Missing values were coded as a separate category and were excluded from test for heterogeneity.

TDF, tenofovir disoproxil fumarate; S-Cr, serum creatinine; CI, confidential interval; HR, incident rate ratio; NNRTI, non-nucleoside reverse transcriptase inhibitor; PI, protease inhibitor; ARV, antiretroviral agent; IDV, indinavir; ART, antiretroviral therapy.

## Discussion

In this study, we evaluated the frequency of S-Cr monitoring during ART in a large cohort of adult HIV-infected individuals in the Asia-Pacific region and described the incidence and prevalence of renal dysfunction after starting TDF. We found S-Cr was assessed at least once a year during ART and more often after starting TDF. Our study also revealed that 4.2% of individuals experienced eGFR decline to <60 ml/min/1.73m^2^ with >30% reduction from baseline or had stopped ART for reported TDF-nephrotoxicity with an incidence of 1.75/100PYs. These data support the need for renal function monitoring for patients receiving TDF-containing ART in resource-limited settings.

Previously, a study from Africa showed limited improvement of prognosis in patients receiving regular laboratory monitoring during ART [19]. The WHO currently does not recommend baseline or routine monitoring of S-Cr during ART in resource-limited settings, in accordance with the results of the DART trial [9]. However, S-Cr was measured regularly in our study sites regardless of country income levels. The possible reason for such routine S-Cr measurement is that the local ART guideline in each country recommends TDF dose reduction when CrCl is <50 ml/min and TDF switch when CrCl is <30 ml/min. Even if S-Cr monitoring is not routinely recommended in the guidelies, the indications to modify TDF dosage may encourage physicians to measure S-Cr regularly. Our data provides an important aspect to the feasibility of regular S-Cr monitoring during TDF use in the Asia-Pacific region.

We observed 1.75/100PYs incidence and 4.2% prevalence of renal dysfunction during a median 2.07 years on TDF. The incidence and prevalence in our study were comparable to previous studies [19–23]. However, while severe renal dysfunction that requiring TDF discontinuation was rare in most of those studies, 13.6% (14/103) of the patients defined as having renal dysfunction discontinued TDF for the reason of TDF-nephrotoxicity. TDF tends to be discontinued by physicians earlier than the guidelines indicate (CrCl 30 ml/min) to avoid making the renal dysfunction irreversible and deciding to discontinue TDF may be easier if other ARV options are available. The severity and the reversibility of renal function need to be considered before deciding to discontinue TDF. Therefore, more information is needed to establish an optimal indication for TDF discontinuation in order to preserve future renal function and minimize unnecessary ARV changes from a long-term perspective.

We found that older age (>50 years), lower pre-TDF eGFR (<60 ml/min/1.73m^2^) and PI-based regimen are associated with higher risk of renal dysfunction in multivariate analysis, all of which were compatible with previous studies and the WHO recommendations for regular S-Cr monitoring [10,19–21,24]. The higher risk in older age and lower pre-TDF eGFR can be explained by the fact that the CKD prevalence rises with age due to the increasing prevalence of risk factors for CKD, such as diabetes and hypertension, and the natural decline of renal function associated with aging [25]. For patients on PI-based regimens, the majority of them (48%) used lopinavir-ritonavir (LPVr). Concurrent use of ritonavir and TDF could result in accumulation of TDF and lead to higher risk for TDF-induced nephrotoxicity [24]. Since LPVr has been widely used as the second line ART for those who failed the first NNRTI-based ART, TDF-nephrotoxicity may be a cause of concen in that population. We did not find an increased risk in those who had lower CD4 counts, higher plasma viral loads or lower body weight, although those factors are reported to predict renal dysfunction during ART [19–23]. The patients in our cohort had lower CD4 counts at ART initiation than those in resource-rich countries and we were not able to compare the risks with a group of high baseline CD4 counts. Since there has been a global shift toward early ART initiation at higher baseline CD4 counts, there may be a limitation in the generalization of our results in this context. In addition, details of the baseline viral loads in almost half of our patients and body weight in 35% were unavailable, which may have affected the power to detect association with renal dysfunction risk. Our results may be useful when we target populations who have more benefit from S-Cr monitoring in resource-limited settings.

Limitations of our study include difficulty in capturing all renal events. Many of the study sites are in resource-limited settings, where the national ART guidelines recommend TDF switch by single test result, and S-Cr was measured approximately twice yearly. Thus, we were not able to utilize secondary confirmatory testing or distinguish between acute and chronic renal dysfunction. In addition, results of urinalysis, electrolytes or other laboratory data to assess tubular function were not collected in TAHOD. These limitations may have affected the classification of renal dysfunction events, which could lead to the underestimation of the renal dysfunction in this study. Moreover, our definition of eGFR decline meant that patients who did not have an eGFR below 60 ml/min/1.73m^2^ were not counted as having renal dysfunction even if the decline was at least 30%. Using this definition could lead to the underestimation of renal dysfunction, however we believe it better relates to the need for TDF discontinuation than the 30% reduction alone. Furthermore, it was not possible to confirm that all renal dysfunction events observed in the study were related to TDF toxicity. Concomitant medications and other drugs that could affect renal function were not collected. Therefore, our results should be interpreted with care.

## Conclusion

In conclusion, 4.2% of individuals on TDF—containing ART experienced renal dysfunction defined as eGFR decline to <60ml/min/1.73m^2^ with >30% reduction from baseline and age older than 50 years, pre-TDF eGFR <60 ml/min/1.73m^2^ and PI-based regimen were at greater risk. These data suggest potential benefits in these higher risk groups for renal function monitoring during TDF-containing ART in resource-limited settings. Further studies are needed to assess the optimal interval of renal function monitoring during TDF use.

## Supporting Information

S1 Table Baseline characteristics of TAHOD patients who have ever received TDF by country. TDF, tenofovir disoproxil fumarate; cART; combination ART, antiretroviral therapy; IQR, interquartile range; AIDS; acquired immune deficiency syndrome; N/A, not applicable.
(XLSX)

S2 Table List of institutional review boards in the study sites. IRB, institutional review board
(XLSX)
